# Longitudinal Associations of Active Versus Passive TikTok Use with Depression: A Chain Mediation Model of Autistic Traits and Self-Esteem

**DOI:** 10.3390/bs16081296

**Published:** 2026-07-29

**Authors:** Hongwei Li, Xiaopan Xu, Cheng Mao, Qingqi Liu

**Affiliations:** 1 School of History and Culture, Henan Normal University, Xinxiang 453007, China; 2013042@htu.edu.cn; 2 School of Sociology, Central China Normal University, Wuhan 430079, China; 3 Institute for Public Policy and Social Management Innovation, College of Political Science and Public Administration, Henan Normal University, Xinxiang 453007, China; 4 Research Center for Urban-Rural Integration Development, Hubei Academy of Social Sciences, Wuhan 430007, China; maocheng2026@126.com; 5 Bay Area School of Applied Psychological Sciences, Beijing Normal University at Zhuhai, Zhuhai 519087, China; liuqingqi@bnu.edu.cn; 6 College of Education for the Future, Beijing Normal University at Zhuhai, Zhuhai 519087, China

**Keywords:** active TikTok use, autistic traits, depression, longitudinal study, passive TikTok use, self-esteem

## Abstract

The global popularity of short-form video applications such as TikTok has increased rapidly. However, longitudinal evidence on how distinct usage patterns affect mental health outcomes, particularly depression, remains limited. The present study investigated whether active versus passive TikTok use differentially predicts depression through the mediating roles of autistic traits and self-esteem. A three-wave longitudinal survey was conducted in China at six-month intervals. A total of 590 university students (aged 17–24 years) completed questionnaires assessing active/passive TikTok use and depression at Time 1 (T1), autistic traits and self-esteem at Time 2 (T2), and depression again at Time 3 (T3). Mediation analyses were performed using PROCESS macro (Model 6), controlling for gender, age, daily TikTok usage time, and T1 depression. Active TikTok use indirectly predicted lower depression through increased self-esteem, but not through autistic traits. In contrast, passive TikTok use predicted higher depression via three distinct pathways: through elevated autistic traits, through reduced self-esteem, and through the serial mediation of autistic traits leading to reduced self-esteem. Active and passive TikTok use exhibit divergent longitudinal associations with depression, mediated by distinct socio-cognitive mechanisms. These findings highlight the importance of distinguishing between usage modes when evaluating the mental health implications of short-video platforms.

## 1. Introduction

Depression is one of the most prevalent mental health problems worldwide (Ibrahim et al., 2013). A random-effects meta-analysis determined that the aggregate point and lifetime prevalence of depression were 12.9% and 10.8%, respectively. The COVID-19 pandemic has exacerbated depression among young individuals (C. Wang et al., 2021). The severity of depression, both before and after the pandemic, may be significantly influenced by individuals’ Internet use behaviors (Kvintova et al., 2024; Santander-Hernández et al., 2022). Several studies have highlighted increased Internet usage, heightened reliance on mobile phones, and augmented dependence on social media among young individuals (Jonnatan et al., 2022; Li et al., 2021; P. Wang et al., 2018). Previous studies have confirmed the effects of different social networking sites (SNSs), such as Facebook, Twitter, and Instagram, on depression (Appel et al., 2016; Huang, 2022; Jeri-Yabar et al., 2019; Khalil et al., 2022). The relationship between TikTok use and mental health, particularly problematic TikTok use and its association with depression, has also attracted significant research attention (Rogowska & Cincio, 2024; Sha & Dong, 2021; N. Yao et al., 2023). For example, a cross-sectional study conducted by Rogowska and Cincio (2024) demonstrated that problematic TikTok use not only directly predicted depression, but also indirectly affected depression among young adults through the mediating effect of procrastination. Similarly, N. Yao et al. (2023) found that depression significantly predicts problematic TikTok use directly and can exert an indirect influence through the mediating role of distress intolerance.

Despite these findings, two notable research gaps warrant further investigation. First, although previous research has examined the relationship between TikTok use and depression, it has predominantly focused on problematic TikTok use, overlooking the potential effects of different usage behaviors, such as active and passive use, on the TikTok user experience. Focusing on specific behaviors associated with TikTok, short video usage is essential for elucidating the psychological effects and mechanisms associated with various usage patterns. Second, previous studies primarily employed cross-sectional questionnaire designs, which limited their ability to rigorously establish predictive relationships between variables, particularly concerning long-term effects. It is crucial to use a longitudinal tracking design to mitigate the challenges associated with data collection at a single time point, thereby providing more robust insights into the long-term effects of short video use. Consequently, we conducted a one-year longitudinal study to analyze the long-term predictive effects of two distinct TikTok use behaviors (active and passive use) on depression, along with their underlying mechanisms.

The introduction is organized to progressively develop the research rationale. It begins by establishing the direct link between distinct patterns of TikTok use and depression, distinguishing between active and passive forms of engagement (Section 1.1). Building on that foundation, the review then turns to the mediating mechanisms that may explain these differential effects. Specifically, autistic traits are examined as a potential socio-cognitive mediator (Section 1.2), followed by self-esteem as a self-evaluative mediator (Section 1.3). The subsequent section integrates both mediators into a sequential chain, exploring whether autistic traits may influence depression via their impact on self-esteem (Section 1.4). Finally, the present study is introduced, summarizing the research questions and the hypothesized model (Section 1.5).

### 1.1. Two Types of TikTok Use Behaviors and Depression

Short-form video applications, such as TikTok, have become popular Internet applications in numerous countries (Chao et al., 2023; D. Zhang et al., 2024). Young individuals constitute a significant group of users of short-form video applications. According to a recent report (Aslam, 2024), approximately 17% of TikTok’s audience comprises individuals aged 18–24 years. A survey conducted among 11,267 Chinese university students during the COVID-19 pandemic revealed that approximately 70% of young individuals in universities spent at least one hour per day on average consuming short-form videos, with approximately 30% dedicated to more than two hours per day (China Youth Daily, 2022). University students frequently watch and post various short-form videos. In China, the use of short-form video applications has adverse effects on the physical and mental health of young individuals (China Internet Network Information Center, 2023); however, the effects of different short-form video use behaviors may vary (Wu et al., 2021).

Drawing on previous studies on the classification of SNS use behaviors (Valkenburg et al., 2022; Verduyn et al., 2022), engagement with short-form videos, such as TikTok, can be categorized into two distinct types: active and passive. Active short-form video use represents typical information generation behavior, whereas passive short-form video use constitutes a form of information browsing behavior (Pan et al., 2023). It is important to note that active use encompasses more than just creating original content. It includes various participatory behaviors, such as sharing others’ content, engaging with videos through likes and comments, and participating in application-based social interactions. Although passive consumption of entertainment and information remains the dominant TikTok usage pattern, active use behaviors, including content creation, sharing, and social interaction, are still present. This is particularly evident among Chinese users (Q. Liu et al., 2025; Pan et al., 2023), in which activities such as posting original content and platform-based socialization represent meaningful, although less frequent, usage patterns compared with passive viewing. Previous studies have found that active use of SNSs is associated with greater life satisfaction (Lian et al., 2020), more positive emotions (de Vaate et al., 2020), and lower levels of depression (Twomey & O’Reilly, 2017). However, passive use of SNSs is negatively associated with subjective well-being (W. Chen et al., 2016) and is linked to negative emotions, such as fear of missing out (Burnell et al., 2019) and depression (H. Z. Wang et al., 2019). Drawing on considerable empirical evidence, Verduyn et al. (2017) put forward the active–passive model of SNS use, which associates active use with positive outcomes and passive use with negative ones. Their framework provides a basis for hypothesizing that active short video use could reduce depression, while passive use might increase it.

Beyond the active–passive model, empirical research also provides evidence relevant to the relationship between specific short-video behaviors and depression. Studies on TikTok have shown that active use is associated with higher self-esteem, whereas passive consumption is linked to lower self-esteem (Pan et al., 2023). A recent longitudinal study found that active short-video use negatively predicted anxiety one year later, while passive use positively predicted it. Notably, both active and passive use negatively predicted self-concept clarity six months later, which in turn mediated their effects on subsequent anxiety (Q. Liu et al., 2025). Regarding depression specifically, recent longitudinal studies have examined problematic short-video use (Qu et al., 2024; D. Zhang et al., 2024), though they have not systematically distinguished between different usage behaviors. For example, short-video overuse positively predicted depression two months later, whereas depression did not significantly predict subsequent overuse (D. Zhang et al., 2024). In summary, while direct longitudinal evidence on active and passive TikTok use in relation to depression remains limited, findings concerning their links to anxiety and the link between problematic use and depression offer relevant support. Based on the existing research, we hypothesize that active and passive TikTok use may show differential associations with depression, with passive use potentially linked to elevated risk and active use potentially linked to reduced risk.

### 1.2. Mediating Role of Autistic Traits

Autistic traits refer to personality characteristics widely distributed in the general population that resemble the core features of autism spectrum disorder (ASD), including social communication difficulties, cognitive patterns, and emotional processing characteristics (Constantino et al., 2006; Constantino & Todd, 2003; Ronald & Hoekstra, 2011). Importantly, autistic traits are conceptually distinct from clinical ASD: the former represents a dimensional construct continuously distributed across the general population, whereas the latter constitutes a diagnostic category defined by clinically significant impairment (Bishop, 2021; Colizzi et al., 2022). Some researchers have conceptualized autistic traits as a potential sixth personality dimension that exists alongside the Big Five personality traits (Wakabayashi et al., 2006). The dimensional perspective represents a paradigm shift from traditional dichotomous conceptualizations of ASD, encouraging quantitative approaches in both clinical practice and scientific research (Guan & Zhao, 2015). The dimensional investigation of autistic traits has emerged as a major research direction in studies on autism, receiving increasing scholarly attention and empirical support (El-Bouhali-Abdellaoui et al., 2025; Gurbuz et al., 2024; Moore et al., 2024; Smees et al., 2024). Autistic traits are significant risk factors for depression. Studies focusing on children and adults have consistently found that autistic traits have a significant positive predictive effect on depression (Ishizuka et al., 2022; Rai et al., 2018). The mechanisms that contribute to the effects of autistic traits on depression may involve school social capital, social problem-solving abilities, and past teasing experiences (Mori et al., 2022; Rosbrook & Whittingham, 2010).

Autistic traits, while showing moderate stability over time, are not immutable. Although personality traits generally exhibit a moderate level of stability, they can undergo change, particularly when subjected to significant environmental influences over extended periods. A substantial body of longitudinal research has shown that personality traits, such as the Big Five factors (Atherton et al., 2021; Roberts et al., 2006), trait optimism (Chopik et al., 2020; Tetzner et al., 2024), and autistic traits (Pender et al., 2020, 2023), can undergo considerable changes even after reaching adulthood. In relation to the manifestation of autistic traits, the empathizing–systemizing theory posits that individual differences in empathic abilities and systemizing tendencies contribute to the expression of autistic characteristics (Baron-Cohen, 2009, 2010). Specifically, when individuals struggle to comprehend others’ emotions and respond appropriately in social contexts while relying extensively on systemizing abilities for pattern-based reasoning, they may display more pronounced autistic-like tendencies. Consequently, environmental factors that significantly affect empathy and systemizing abilities may lead to variations in the expression of autistic traits. The context of the COVID-19 pandemic and its aftermath provides a valuable lens for examining the link between TikTok use and autistic traits. Prevailing social restrictions and isolation are theorized to have exerted direct effects on such traits while concurrently heightening individuals’ reliance on and vulnerability to social media influences. According to the active–passive model of social network site use (Verduyn et al., 2017), active and passive short-video use are likely to have distinct associations with autistic traits.

Passive browsing behavior, characterized by non-interactive viewing of algorithmically recommended content, may be associated with cognitive-behavioral patterns related to autistic traits through several hypothesized mechanisms. First, an attentional mechanism may be involved. Short-video platform algorithms tend to recommend content that aligns with a user’s past preferences, often featuring high repetition and strong patterning. Long-term passive immersion in such an environment may reinforce an individual’s attentional bias toward systematic, predictable information while diminishing attention to and interest in complex, unstructured social cues (Y. Chen et al., 2023; Wu et al., 2025). Second, affective processing may be affected. Passively consumed, high-volume, fragmented video content often lacks the emotional context inherent in real social interaction. Frequent passive viewing may reduce opportunities for practicing the rapid identification, integration, and response to subtle emotional signals from others, potentially leading to emotional overload and thereby affecting affective processing (X. Wang et al., 2025). Third, a social-cognitive mechanism may be involved. Browsing behavior lacking bidirectional interaction fails to provide practice opportunities for reasoning about social intentions and interpreting mental states. Over time, individuals may become increasingly accustomed to offering superficial, rule-based “systemizing” explanations for others’ behaviors, rather than employing empathic, “mentalizing” understanding. According to the empathizing–systemizing theory of autism (Baron-Cohen, 2009, 2010), frequent exposure to algorithmically curated short videos featuring common patterns may encourage a systemizing cognitive style. Such exposure could lead individuals to abstract and generalize about people and events rather than analyzing their unique characteristics in detail. Excessive systemizing tendencies may render certain thoughts and behaviors increasingly patterned and rigid. Based on the mechanisms outlined above, we hypothesize that passive short-video use may be positively associated with autistic trait levels, potentially through reinforcing a systematic attentional bias, reducing practice in emotional resonance, and solidifying non-mentalizing social cognitive patterns. However, we acknowledge that these proposed mechanisms were not directly measured in the present study and that the observed effects, if any, are likely to be modest in magnitude given the relative stability of autistic traits. The findings should therefore be interpreted as preliminary evidence consistent with, rather than definitive proof of, the hypothesized pathways.

In contrast, active use behaviors, such as creating and sharing content to seek connection, may provide a social environment that could be associated with the aforementioned vulnerabilities through several mechanisms. First, active short-video use may relate to attentional and cognitive patterns. To create resonant content, individuals need to actively engage with social trends and understand others’ perspectives, thereby shifting attention from the self or fixed interests toward broader social information. Second, active use may relate to emotional expression and regulation. Sharing personal experiences or viewpoints involves constructing emotional narratives and anticipating or interpreting audience feedback, serving as potential practice for emotional expression and online socio-affective skills. Third, active use may enable social feedback learning. The social feedback received after posting, such as likes and comments, provides a low-threat environment for individuals to practice interpreting ambiguous social signals (e.g., silence, brief comments) and adjusting social behavior. Improvements in emotional intelligence, empathy, and social skills have been associated with reductions in autistic traits (Higuchi et al., 2017; Koegel et al., 2016; Tonge et al., 2016). Therefore, we hypothesize that active short-video use may be negatively associated with autistic trait levels, potentially by providing mentalizing practice, facilitating emotional expression and regulation, and offering positive social feedback learning.

### 1.3. Mediating Role of Self-Esteem

Low self-esteem has been consistently associated with elevated depressive symptoms in both adolescent and adult populations (Orth et al., 2009; van Tuijl et al., 2014). According to the cognitive model of depression, dysfunctional schemas, which specifically involve negative self-attitudes and biases, are central to the onset and maintenance of depression (Beck, 2008). Self-esteem, as a core reflection of these self-related cognitions, has consequently been a major focus of empirical research, with substantial evidence supporting its predictive role in depression (Sowislo & Orth, 2013; Tehrani et al., 2025). Lower levels of self-esteem not only show direct associations with negative emotions and increased depression risk (Nima et al., 2013; Salavera et al., 2020), but also appear to intensify the effect of negative environmental factors on depressive symptoms (Jiang et al., 2021; Meier et al., 2009). A comprehensive meta-analysis of longitudinal studies further substantiates the significant negative long-term predictive relationship between self-esteem and depression (Sowislo & Orth, 2013). Collectively, these findings offer a compelling empirical basis for the critical link between self-esteem and depression, underscoring its public health relevance.

Sociometer theory (Leary, 2005) provides a theoretical framework for understanding how social media use may be associated with self-esteem. According to sociometer theory, self-esteem functions as a psychological gauge of perceived social acceptance and belonging. When individuals perceive rejection or devaluation from others, their self-esteem tends to decline. Active TikTok use, as an information generation behavior, allows individuals to selectively present an ideal self-image or a positive life (Du et al., 2022). Actively sharing short-form videos can help individuals receive positive feedback. Both positive self-presentation and social feedback have been associated with increased self-esteem among young individuals (Han & Yang, 2023; Meeus et al., 2019). In contrast, passive TikTok use may place individuals in environments with abundant social comparison cues. Because of the prevalence of overly positive information on short-form video platforms, many young individuals may engage in upward social comparisons when watching short-form videos (Wu et al., 2021). Comparing oneself with someone who appears better off in some aspects may lead to increasingly negative self-evaluations (Han & Yang, 2023; Jiang & Ngien, 2020). Empirical research has shown that passive TikTok use is negatively correlated with appearance and weight esteem, whereas active engagement is positively associated with these measures (Pan et al., 2023). Therefore, self-esteem may play a mediating role in the relationship between the two types of TikTok use behaviors and depression. Active TikTok use may be negatively associated with depression through the mediating effect of self-esteem. Conversely, passive TikTok use may demonstrate a positive association with depression, mediated by self-esteem. However, we acknowledge that these proposed pathways reflect theoretical predictions derived from the literature rather than empirically established causal sequences.

### 1.4. Chain Mediating Role or Autistic Traits and Self-Esteem

When considering autistic traits and self-esteem as mediators in the relationship between TikTok use and depression, these two constructs may not function independently but rather operate sequentially. Existing studies indicate that individuals with ASD and elevated autistic traits exhibit pronounced negative attentional bias, characterized by increased sensitivity to negative stimuli and a tendency to interpret neutral or positive stimuli negatively (South et al., 2022; Unruh et al., 2020; X. Zhang et al., 2024). Studies have demonstrated that autistic traits exert direct and indirect reinforcing effects on negative emotions (Zhao et al., 2020; Zhao et al., 2021). According to the broaden-and-build theory of positive emotions (Fredrickson, 2004), positive emotions expand cognitive and behavioral repertoires, thereby facilitating the construction of enduring psychological and social resources. In contrast, negative emotions tend to narrow these cognitive-behavioral options and impede resource development. Substantial empirical evidence supports the notion that positive emotions enhance self-esteem, whereas negative emotions significantly undermine it (Y. Liu et al., 2014; Rodrigues et al., 2022). Consequently, individuals with elevated autistic traits may be at a heightened risk of developing lower self-esteem, potentially due to negative attentional bias and the experience of negative emotions.

Direct empirical evidence supports the proposed links. Research indicates that children and adolescents with autism spectrum disorder are at elevated risk for low explicit self-esteem, which frequently co-occurs with depressive symptoms (McCauley et al., 2019; van der Cruijsen & Boyer, 2021). Even in the general population, autistic-like traits in childhood have been found to modestly but significantly predict later internalizing symptoms (Hallett et al., 2010). Most directly relevant, a longitudinal study confirmed an indirect pathway from autistic traits to later anxiety and depression via reduced self-compassion in both autistic and non-autistic adults (Galvin et al., 2024). Collectively, these findings are consistent with a conceptual chain wherein autistic traits are associated with vulnerabilities in self-evaluation (e.g., low self-esteem), which in turn show associations with depression. Extending that reasoning to the social media context, specific patterns of TikTok use may be associated with autistic traits, which may in turn relate to lower self-esteem, and ultimately show associations with higher depression levels. Nevertheless, the proposed chain mediation pathway should be interpreted as a theory-driven hypothesis that has received preliminary empirical support, rather than as a definitive causal sequence.

### 1.5. The Present Study

The COVID-19 pandemic and its aftermath brought issues related to short-video use and mental health into sharper focus (Mousoulidou et al., 2024; Y. Yao et al., 2025). The widespread social restrictions characterizing that period should not be viewed merely as isolated confounding variables; rather, they serve as a “societal stress-testing ground” that amplifies ever-present risks, including excessive reliance on online interaction when offline social resources are scarce and heightened psychological reactivity to social media content under stress. Importantly, these challenges have not dissipated with the pandemic’s end, but have evolved into sustained concerns with a stable presence across diverse groups. Moreover, the state of constrained social connectedness persists in multiple forms, including recurrent public health advisories urging reduced gatherings and the chronic social isolation experienced by vulnerable populations. For these groups, short-video platforms often function as critical channels for obtaining information, seeking companionship, and constructing alternative social support. Thus, the post-pandemic context provides a valuable lens for examining the relationships between short-video use and mental health, and the present study capitalizes on that opportunity by investigating these dynamics in a longitudinal sample.

**Figure 1:**
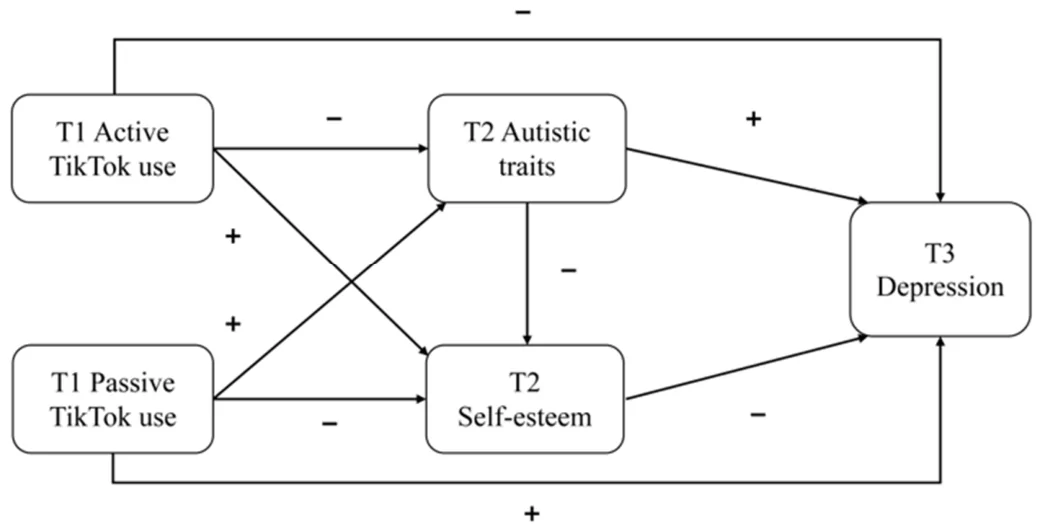
The hypothesized mediation model.

The present study examined the associations and potential pathways linking two types of TikTok use behavior (active and passive) to depression among young individuals. Specifically, we investigated the direct association between TikTok use and depression, as well as its indirect association through autistic traits and self-esteem. To test our hypothesized model, we employed a one-year longitudinal questionnaire design and conducted a three-wave study to examine whether baseline TikTok use (T1) was associated with autistic traits and self-esteem six months later (T2), which in turn were associated with depression six months later (T3). Figure 1 presents the hypothesized mediation model. The proposed model draws on and integrates multiple classic theories into a coherent framework that follows a logical sequence: from behavioral input (use behavior) to the development of stable cognitive vulnerabilities (personality traits → self-evaluation), and ultimately to emotional health outcomes. Drawing on the previous evidence, we propose the following hypotheses:

**Hypothesis** **1.** *T1 active TikTok use is negatively associated with T3 depression.*

**Hypothesis** **2.** *T1 passive TikTok use is positively associated with T3 depression.*

**Hypothesis** **3a.** *T2 autistic traits mediate the relationship between T1 active TikTok use and T3 depression.*

**Hypothesis** **3b.** *T2 autistic traits mediate the relationship between T1 passive TikTok use and T3 depression.*

**Hypothesis** **4a.** *T2 self-esteem mediates the relationship between T1 active TikTok use and T3 depression.*

**Hypothesis** **4b.** *T2 self-esteem mediates the relationship between T1 passive TikTok use and T3 depression.*

**Hypothesis** **5a.** *T2 autistic traits and T2 self-esteem sequentially mediate the relationship between T1 active TikTok use and T3 depression.*

**Hypothesis** **5b.** *T2 autistic traits and self-esteem sequentially mediate the relationship between T1 passive TikTok use and T3 depression.*

## 2. Materials and Methods

### 2.1. Participants

The present study received approval from the Ethics Committee of the College of Education for the Future, Beijing Normal University at Zhuhai (approval number: 310432115, approval date: 26 December 2021). Informed consent was obtained from all individual participants involved in the study. Participants were recruited from two universities in South China through online advertisements on campus forums and in-class announcements during April 2022. Eligibility criteria included: (a) being a current undergraduate student at one of the participating universities, (b) being a user of short-form video platforms (particularly TikTok), and (c) providing informed consent voluntarily. For participants under 18 years of age, consent was obtained from their guardians. Participation was voluntary, and participants received a notebook and a pen following each wave as a token of appreciation. The study employed a three-wave longitudinal design with six-month intervals between assessments. Time 1 (T1) data collection occurred in April 2022, during the “dynamic zero-COVID” policy period in China, when strict social distancing and quarantine measures were in place. Time 2 (T2) occurred in October 2022, when similar restrictions remained in effect. Time 3 (T3) occurred in April 2023, after the relaxation of COVID-19 restrictions, when most social activities had resumed. The temporal sequence allowed us to examine how TikTok use patterns during a period of social restriction predicted socio-cognitive states and mental health outcomes as the social context changed. The initial sample comprised 656 eligible students invited in April 2022. At Time 1, 636 students completed the survey (96.9% response rate). Six months later, at Time 2 (October 2022), 618 students (97.2% of the initial sample) completed the second survey. At Time 3 (April 2023), the sample was reduced to 590 students, representing 92.8% of the initial sample. Given that the number of participants with missing values in our sample was extremely small, and that for each such participant the proportion of missing questionnaire items was also minimal (less than 1%), we employed mean imputation to handle the missing data. When the proportion of missing data is very low, the results obtained from various missing data handling methods are highly comparable. Comparisons between participants who completed all three waves and those who dropped out revealed no significant differences in baseline demographic characteristics or key study variables (all *ps* > 0.05), suggesting that attrition bias is unlikely to have substantially influenced the findings. Data from 590 university students (287 women and 303 men) were included in the formal analysis to test the hypotheses. Participants’ ages ranged from 17 to 24 years, with a mean age of 19.83 years (*SD* = 1.24).

### 2.2. Measurements

The study employed a three-wave longitudinal design with six-month intervals between assessments. At Time 1 (T1; April 2022), participants completed measures of active and passive TikTok use, as well as the depression scale (the latter serving as a baseline covariate). At Time 2 (T2; October 2022), participants completed measures of autistic traits and self-esteem. At Time 3 (T3; April 2023), participants completed the depression scale again as the outcome measure. The staggered measurement schedule was adopted to reduce participant burden and minimise attrition across the three waves.

#### 2.2.1. TikTok Use Behaviors

Our measurement items were adapted from the established active–passive social media use framework (e.g., Verduyn et al., 2017; Frison & Eggermont, 2016), which has been widely validated across multiple social media platforms. In the questionnaire instructions, participants were explicitly asked to report their short-form video use behaviors on TikTok (or Douyin, the Chinese version of TikTok). Active TikTok use were measured using three items (e.g., “I post short-form videos about my life publicly”) adapted from the Active Facebook Use Scale (Frison & Eggermont, 2016); Passive TikTok use was measured using three items (e.g., “I scroll through the news feed of short-form videos without engaging in commenting or reposting”) adapted from the Chinese version (Q. Liu et al., 2017) of the passive SNS use scale (Tandoc et al., 2015). These items are rated on a seven-point scale (1 = almost never, 7 = almost always). Higher scores on the two scales indicated stronger engagement in active and passive TikTok use behaviors. The items were also specifically reworded to refer to TikTok use behaviors rather than general social media use. For active use, items assess content creation, sharing, and social interaction (e.g., “post short-form videos”); for passive use, items assess browsing and consumption without interaction (e.g., “view short-form videos created by others”). These behaviors are theoretically and operationally distinct and are consistent with the conceptualizations used in prior TikTok research (e.g., Pan et al., 2023; Q. Liu et al., 2025). The original items were developed in English and then translated into Chinese using a standard forward-backward translation procedure. Specifically, the scale was first translated from English to Chinese by two bilingual researchers independently, with any discrepancies resolved through discussion. The resulting Chinese version was then back-translated into English by a third bilingual translator who was blind to the original version. Finally, the back-translated version was compared with the original to ensure conceptual equivalence. The forward-backward translation procedure was conducted with reference to the guidelines recommended by Brislin (1970).

Both subscales demonstrated good internal consistency in the present sample (active use: α = 0.93; passive use: α = 0.90). To assess the construct validity of the adapted scales, confirmatory factor analyses (CFA) were conducted for the active and passive use subscales separately. The CFA results for the active use scale indicated a good model fit: *χ*^2^/*df* = 3.71, RMSEA = 0.05, CFI = 0.96, TLI = 0.95, and SRMR = 0.04. For the passive use scale, the CFA results also demonstrated a good fit: *χ*^2^/*df* = 2.99, RMSEA = 0.04, CFI = 0.97, TLI = 0.97, and SRMR = 0.03. These findings suggest that both the active and passive short-video use scales possess satisfactory construct validity.

#### 2.2.2. Autistic Traits

Autistic traits were assessed using the Chinese Short Version of the Autism Spectrum Quotient (Hu, 2021). The gold standard for quantitative assessment of autistic traits is the Autism Spectrum Quotient (AQ), originally developed by Baron-Cohen et al. (2001). AQ exists in multiple validated versions, including adult self-report, adolescent proxy-report, and early childhood proxy-report formats, and has been cross-culturally adapted worldwide with demonstrated reliability and validity (Lundqvist & Lindner, 2017; Omelańczuk & Pisula, 2025; Ruzich et al., 2015). The AQ short form has been widely used in non-clinical general population samples and has demonstrated acceptable psychometric properties, including adequate internal consistency and expected sex differences (Ruzich et al., 2015). For Chinese participants, we employed the culturally adapted short version of the AQ, which was translated and validated by Hu (2021) specifically for self-assessment. It comprises 22 items (e.g., “I enjoy social occasions”) rated on a four-point scale (1 = strongly agree, 4 = strongly disagree). Higher scores indicated greater autistic traits. Cronbach’s α was 0.93, suggesting a high level of internal consistency.

#### 2.2.3. Self-Esteem

The Chinese version (X. D. Wang et al., 1999) of the Rosenberg Self-Esteem Scale (Rosenberg, 1965) was adopted, excluding one item (“I wish I could have more respect for myself”) because of potential cultural differences (Q. Liu et al., 2017). Nine items, including “On the whole, I am satisfied with myself”, were rated on a four-point scale (1 = strongly disagree, 4 = strongly agree). Higher scores indicated increased self-esteem. Cronbach’s α for this measure was 0.90.

#### 2.2.4. Depression

Depression was measured using the Depression Subscale of the Chinese version (Gong et al., 2010) of the Depression Anxiety Stress Scale (DASS-21; Lovibond & Lovibond, 1995). Participants rated seven items on a four-point scale (0 = does not apply to me, 3 = applies to me very much or most of the time). An example item is “I felt that I had nothing to look forward to”. Higher scores indicated elevated levels of depression. Cronbach’s α for this measure were 0.92 and 0.91 at Times 1 and 3, respectively.

### 2.3. Statistical Analysis

Pearson’s correlation analysis was conducted to investigate the correlational relationships among variables. Mediation analyses were conducted using PROCESS macro (Model 6) for SPSS Version 26.0 (Hayes, 2018) with 5000 bootstrap resamples to estimate bias-corrected confidence intervals for indirect effects. PROCESS was chosen over structural equation modeling (SEM) for several reasons. First, the staggered measurement design, involving different constructs assessed at different waves, was not optimally suited for a fully specified SEM with all possible cross-lagged paths. Second, the hypothesized model had a clear directional structure, mapping directly onto PROCESS’s regression-based path analysis framework, with T1 use predicting T2 mediators and T2 mediators predicting T3 depression. Third, for observed variable models such as the one used in the study, PROCESS and SEM yield largely equivalent results (Hayes et al., 2017). A sensitivity power analysis using G*Power Version 3.1 indicated that with *N* = 590, α = 0.05, and power = 0.80, our sample was adequately powered to detect small-to-medium effects (*f*^2^ ≥ 0.04). Gender, age, daily duration of TikTok use, and T1 depression were included as covariates.

## 3. Results

### 3.1. Preliminary Analysis

Given that all variables were assessed via self-report measures, we conducted the Harman single-factor test (Podsakoff et al., 2003) to examine the potential threat of common method bias. All items from the key study variables were entered into an exploratory factor analysis. The results showed that 17 factors had eigenvalues greater than 1, and the first factor accounted for 18.19% of the total variance, which was far below the critical threshold of 40%. These results indicate that common method bias is not a significant concern in the present study.

The correlational relationships among the variables are presented in Table 1. T1 active TikTok use was positively associated with T2 self-esteem (*r* = 0.42, *p* < 0.001) and negatively associated with T1 depression (*r* = −0.18, *p* < 0.001), T2 autistic traits (*r* = −0.13, *p* < 0.01), and T3 depression (*r* = −0.30, *p* < 0.001). Conversely, T1 passive TikTok use was negatively correlated with T2 self-esteem (*r* = −0.51, *p* < 0.001) and positively correlated with T1 depression (*r* = 0.24, *p* < 0.001), T2 autistic traits (*r* = 0.43, *p* < 0.001), and T3 depression (*r* = 0.36, *p* < 0.001). Additionally, T2 self-esteem was negatively correlated with T1 depression (*r* = −0.27, *p* < 0.001), T2 autistic traits (*r* = −0.56, *p* < 0.001), and T3 depression (*r* = −0.53, *p* < 0.001). T2 autistic traits had a positive relationship with T1 depression (*r* = 0.22, *p* < 0.001) and T3 depression (*r* = 0.41, *p* < 0.001).

### 3.2. Testing for the Mediation Model

Prior to testing the mediation hypotheses, we examined the assumptions of multicollinearity and normality. Variance inflation factors (VIFs) for all predictors ranged from 1.12 to 3.48, with tolerance values ranging from 0.29 to 0.89, indicating that multicollinearity was not a concern (all VIFs < 5). Normality was assessed by examining the skewness and kurtosis of all study variables. All absolute skewness values were below 1.5 and all absolute kurtosis values were below 3.0, indicating that the normality assumption was reasonably met for the main analyses. Table 2 shows the results of the mediation analysis that examines the roles of autistic traits and self-esteem in the association between active and passive TikTok use and depression. In terms of the association between active TikTok use and depression, results revealed a direct negative relationship between T1 active TikTok use and T3 depression (β = −0.20, *p* < 0.001, 95%CI [−0.27, −0.12]) when controlling for the effects of gender, age, duration of TikTok use, and T1 depression. Additionally, there was no significant prediction of T2 autistic traits by T1 active TikTok use (β = −0.05, *p* = 0.17, 95%CI [−0.13, 0.02]). However, T1 active TikTok use significantly predicted self-esteem (β = 0.32, *p* < 0.001, 95%CI [0.25, 0.39]). Moreover, T2 autistic traits negatively predicted T2 self-esteem (β = −0.38, *p* < 0.001, 95%CI [−0.45, −0.30]). When mediators (i.e., T2 autistic traits and T2 self-esteem) were included in the analysis, T2 autistic traits was positively associated with T3 depression (β = 0.13, *p* < 0.01, 95%CI [0.03, 0.22]), whereas T2 self-esteem was negatively associated with T3 depression (β = −0.29, *p* < 0.001, 95%CI [−0.40, −0.19]). Furthermore, the predictive effect of T1 active TikTok use on T3 depression remained significant (β = −0.09, *p* < 0.05, 95%CI [−0.16, −0.02]) even after accounting for the two proposed mediators (T2 autistic traits and T2 self-esteem).

Regarding the association between passive TikTok use and depression, results showed that higher levels of T1 passive TikTok use were directly associated with an increase in T3 depression (β = 0.23, *p* < 0.001, 95%CI [0.15, 0.31]) when controlling for the effects of gender, age, duration of TikTok use, and T1 depression. Additionally, there was a significant positive prediction of T2 autistic traits by T1 passive TikTok use (β = 0.38, *p* < 0.001, 95%CI [0.29, 0.48]), a negative prediction of T2 self-esteem by T1 passive TikTok use (β = −0.29, *p* < 0.001, 95%CI [−0.37, −0.22]), and a negative prediction of T2 self-esteem by T2 autistic traits (β = −0.38, *p* < 0.001, 95%CI [−0.45, −0.30]). When incorporating these mediators into the analysis, T2 autistic traits was positively associated with T3 depression (β = 0.13, *p* < 0.01, 95%CI [0.03, 0.22]), whereas T2 self-esteem was negatively associated with depression at T3 (β = −0.29, *p* < 0.001, 95%CI [−0.40, −0.19]). Interestingly, when incorporating the proposed mediators (T2 autistic traits and T2 self-esteem) in the model, the predictive effect of T1 passive TikTok use became non-significant (β = 0.06, *p* = 0.12, 95%CI [−0.01, 0.13]).

**Figure 2:**
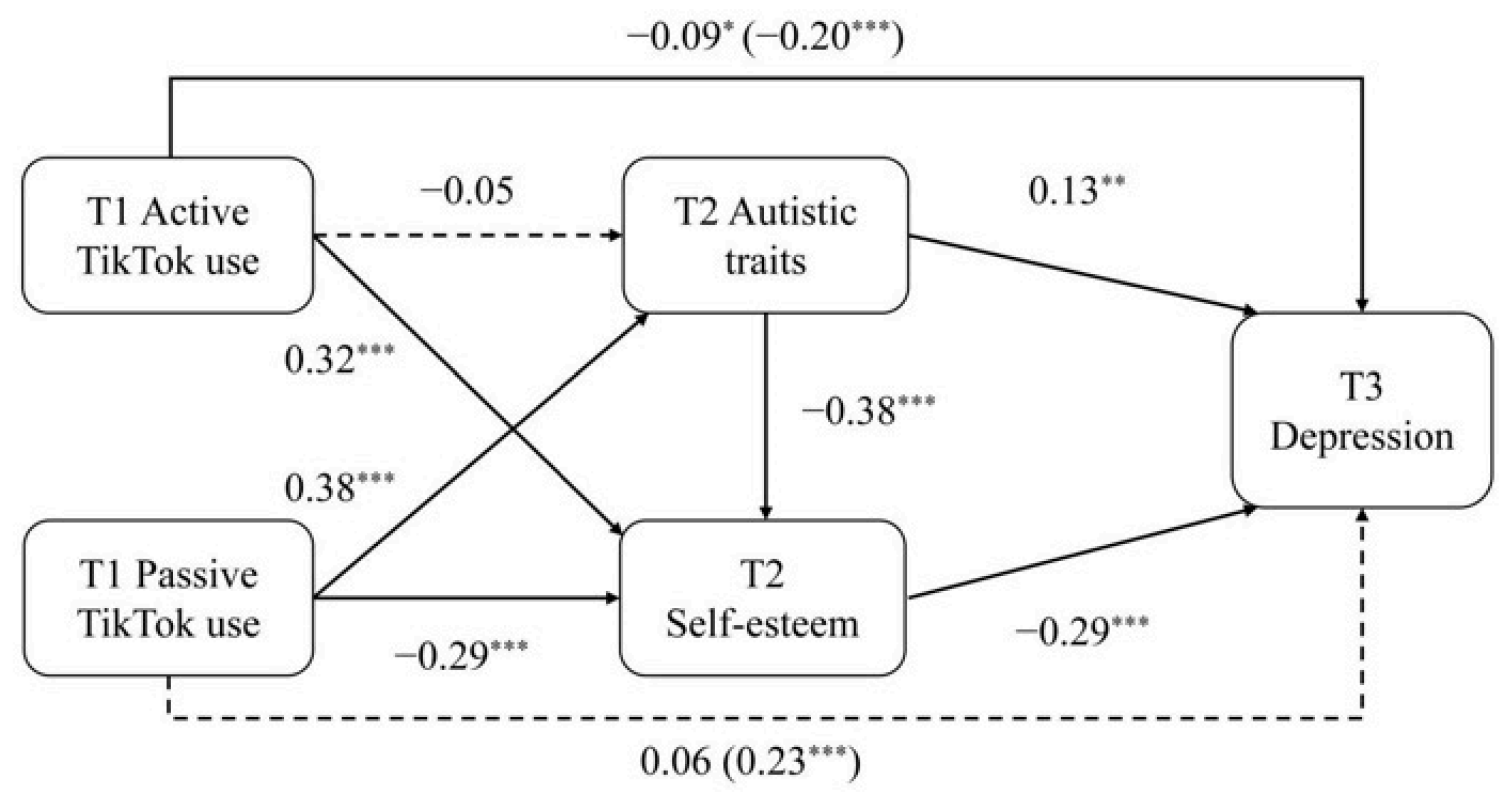
Mediation model of autistic traits and self-esteem in the association between TikTok use and depression. Note. Gender, age, duration of TikTok use, and T1 depression were controlled in the model. The coefficient enclosed in parentheses indicates the value of the direct effect in the absence of the mediator variable. * *p* < 0.05, ** *p* < 0.01, *** *p* < 0.001.

The bias-corrected percentile bootstrap method (Table 3 and Figure 2) revealed that only one indirect path from T1 active TikTok use to T3 depression was statistically significant. The mediating effect of autistic traits and chain mediating effects of autistic traits and self-esteem were not significant. However, the mediating effect of T2 self-esteem was significant. The mediating effect of T2 self-esteem was −0.09, with a 95% confidence interval of [−0.14, −0.05], accounting for 47.94% of the total effect of active TikTok use on depression.

In contrast, three noteworthy indirect pathways associated T1 passive TikTok use with T3 depression, as shown in Table 3 and Figure 2. The mediating effect of T2 autistic traits was 0.05 (95% CI [0.01, 0.09]), accounting for 21.21% of the total effect of passive TikTok use on depression. Similarly, the mediating effect of T2 self-esteem was 0.09 (95% CI [0.05, 0.13]), accounting for 36.67% of the overall effect of passive TikTok use on depression. Furthermore, the chain-mediating effect of autistic traits (T2) and self-esteem (T2) was 0.04 (95% CI [0.02, 0.07]), representing 18.18% of the total effect of passive TikTok use on depression.

## 4. Discussion

The frequent use of social media, particularly short-form video apps such as TikTok, has been a prominent topic among the Chinese population, with much discussion centered on its association with psychological issues and its potential role in mental health problems. Using a one-year longitudinal design, the present study examined the direct and indirect effects of two types of TikTok use behavior on depression among emerging Chinese adults. To the best of our knowledge, the study is one of the earliest to explore the effects and underlying mechanisms of diverse TikTok use patterns on depression. The present findings not only confirm the longitudinal relationship between active and passive TikTok use and depression but also reveal the internal pathways that may underlie that relationship. These results contribute to a better understanding of the existing body of research on the association between Internet use and depression. These findings provide educators and parents with practical guidance for supporting young individuals, particularly those experiencing social isolation or heightened social stress, when using short-form video applications.

### 4.1. Direct Effects of Two Types of TikTok Use Behavior on Depression

The findings offer empirical support for Hypothesis 1 and Hypothesis 2. Specifically, the results confirmed Hypothesis 1 by revealing an inverse relationship between active TikTok use and depression while concurrently supporting Hypothesis 2 through the identified positive association between passive TikTok use and depression. These contrasting patterns suggest that different modes of engagement with TikTok are associated with various relationships with depression. These results are consistent with those of previous studies that demonstrated a positive correlation between the passive use of SNSs and depression, as well as a negative correlation between the active use of SNSs and depression (Valkenburg, 2022; Verduyn et al., 2022). Nevertheless, our study contributes to that body of knowledge by specifically focusing on the use of TikTok, a prevalent short-form video application in the current mobile Internet landscape. The associations between short-form video applications and mental health are complex, with distinct patterns of association linked to various forms of use. Even in the social environment of the COVID-19 pandemic, short-form video applications showed a range of outcomes beyond simply positive or negative ones. Given that complexity, in daily life, it is important to differentiate the specific patterns of association linked to different TikTok use behaviors.

### 4.2. Mediating Effect of Autistic Traits

The results support Hypothesis 3b but do not support Hypothesis 3a. Elevated autistic traits mediated the association between passive TikTok use and increased depression. However, decreased autistic traits did not mediate the relationship between active TikTok use and reduced depression. Consistent with previous research on autistic traits and depression (Ishizuka et al., 2022; Rai et al., 2018), our findings showed that autistic traits were significantly associated with an elevated risk of depression. There are two possible explanations for the association between autistic traits and depression. First, individuals with higher levels of autistic traits may struggle with problem-solving skills (Einadab et al., 2022) and are less likely to receive social support, which is crucial for resolving issues (Mori et al., 2022; S. Zhang et al., 2024). Consequently, they may experience heightened depression when confronted with negative events. Second, these individuals may face greater challenges in describing and expressing their emotions (Vuillier et al., 2020). Such difficulties not only hinder the appropriate release of negative emotions but also result in the continuous accumulation of negative feelings, ultimately increasing the risk of depression (Hemming et al., 2019; Sagar et al., 2021). Contrary to our hypothesis, active TikTok use did not show a significant association with autistic traits. That null finding suggests that active engagement on TikTok, such as posting and sharing content, may be insufficient to produce observable changes in socio-cognitive traits like autistic-like tendencies in a general population sample over a six-month period. One possible explanation is that active use, despite its socially engaging nature, may not substantially affect the attentional and socio-cognitive patterns associated with autistic traits. An alternative account is that longer exposure may be required for such associations to become detectable.

When comparing the patterns of association for passive versus active TikTok use, only passive use was significantly associated with subsequent autistic traits in our data. Specifically, the identified longitudinal pathway aligns with prior evidence linking problematic short-video use to attention difficulties. As platform algorithms habitually recommend preference-aligned content, attention may become increasingly channeled toward exaggerated, pattern-based features, thereby reinforcing a systematic attentional bias. Furthermore, passive SNS use, characterized by non-reciprocal browsing, is associated with diminished social interaction, impoverished emotional communication, and attenuated social skills (Brown, 2013; Aoun, 2014). Most critically, frequent, algorithm-driven exposure to homogenized content may promote a rigid, systemizing cognitive style, wherein individuals interpret social information through an overly schematic framework. It is plausible that these converging mechanisms could collectively worsen autistic traits over time, consistent with prior work (Higuchi et al., 2017; Tonge et al., 2016). The significant mediation through autistic traits extends the applicability of the empathy–systemizing theory (Baron-Cohen, 2009, 2010) to the domain of passive social media use. Our findings illustrate a way in which the theory can help operationalize and explain the psychological impact of such behaviors. Moreover, they underscore the value of conceptualizing autistic traits as a multifaceted mediator that captures interrelated vulnerabilities across attentional, emotional, and social-cognitive domains, thereby offering a productive theoretical lens for future research into analogous media effects.

In contrast to the pattern of associations found for passive use, active TikTok use showed no significant longitudinal association with autistic traits in our data. Although active online engagement (e.g., communicating and sharing via short videos) can increase interaction frequency, it may not adequately foster the core socio-cognitive capacities—like nuanced emotional intelligence and complex social skills—that are central to autistic traits. The present result is consistent with the extended active–passive model of social network site use (Verduyn et al., 2022), which posits that the benefits of active use are context-dependent rather than universal and can sometimes yield neutral or adverse outcomes. Collectively, the findings point to a nuanced relationship. Online behaviors carrying clear social risk, such as passive and immersive browsing, show a potent link to exacerbating autistic traits. In comparison, behaviors typically considered positive may not reliably improve this specific aspect of socio-cognitive functioning, even if they confer broader mental health benefits. The socio-restrictive context of the pandemic and its aftermath, which heightened overall social media dependency, likely amplified the impact of passive use in particular. Such passive, algorithm-driven consumption could reinforce rigid cognitive patterns and foster greater social reticence in offline settings. From a practical standpoint, the results underscore the necessity for targeted interventions. Mitigating the risk of worsening autistic traits necessitates a primary focus on reducing prolonged, passive TikTok consumption. However, merely promoting active use without attention to the quality and psychological safety of that engagement is likely insufficient. Consequently, future initiatives should prioritize guiding users toward digital interactions that are more meaningful, interactive, and socially reciprocal. Such forms of engagement are theoretically posited to be more conducive to genuine socio-cognitive growth.

### 4.3. Mediating Effect of Self-Esteem

Furthermore, the results support Hypotheses 4a and 4b. Self-esteem significantly mediates the relationship between two TikTok use behaviors and depression. Our results are consistent with those of previous studies on the relationship between self-esteem and depression (Nima et al., 2013; Salavera et al., 2020). According to the Cognitive Diathesis-Stress Theory of Depression, non-adaptive cognition (e.g., low self-esteem) is a key proximal factor in depression (Abela & Sullivan, 2003; Lakdawalla et al., 2007). Short-form video use behavior, as a distant factor, can affect depression by influencing the proximal factor of self-esteem. In addition, the patterns of association between different TikTok use behaviors and self-esteem differ; watching TikTok videos tends to be linked to lower self-esteem, whereas sharing TikTok videos tends to be linked to higher self-esteem. Upward social comparison induced by passive use (Pan et al., 2023) may be a significant reason for a decrease in self-esteem (Q. Liu et al., 2017; Yoon et al., 2019). However, ideal self-image and positive feedback generated by active use behaviors (Sth & Palupi, 2022) may be key paths for improving self-esteem (Burrow & Rainone, 2017; Yang & Bradford Brown, 2016). These findings tentatively suggest that moderating TikTok use for sharing and communication while limiting excessive consumption may be associated with a more positive self-concept in daily life, though further research is needed to establish the direction and nature of that relationship.

### 4.4. Chain Mediating Effect of Autistic Traits and Self-Esteem

Notably, the study revealed a serial mediation effect of autistic traits and self-esteem in the relationship between passive TikTok use and depression. Specifically, Hypothesis 5a was not supported, whereas Hypothesis 5b was supported. Existing studies suggest that individuals with ASD often experience lower levels of self-esteem (McCauley et al., 2019; van der Cruijsen & Boyer, 2021). Consistent with these findings, we observed a significant association between higher levels of autistic traits and lower self-esteem. Individuals with elevated levels of autistic traits may harbor a negative self-concept (McCauley et al., 2019) owing to the presence of the characteristic symptoms of autism, and they may also be susceptible to negative evaluations from others (Rosbrook & Whittingham, 2010). These factors showed associations with lower self-esteem in our sample. Extending that logic, passive TikTok use may be linked to autistic traits, which in turn may be associated with reduced self-esteem and potentially with higher depression levels. These findings suggest that examining TikTok use in relation to depression may benefit from considering multiple individual factors, though the practical implications of such an approach require further investigation.

Although the indirect effects observed in the present study reached statistical significance, their magnitude was modest. For example, the largest indirect effect accounted for 36.67% of the total effect of passive use on depression, corresponding to an effect of approximately 0.09 on the mediation scale. Effect sizes of that magnitude should be interpreted with appropriate caution. In the context of longitudinal research examining complex psychological processes over six-month intervals, even modest indirect effects can carry theoretical value, as they represent systematic pathways through which social media use may influence mental health. However, the practical significance of those effects, particularly their translation into clinically meaningful changes, remains uncertain and warrants further investigation. We thus interpret the findings as preliminary evidence for the proposed mechanisms rather than as definitive estimates of practical impact.

## 5. Limitations

First, the nature of the sample limits the generalizability of our findings. The sample was drawn from a convenience pool of university students in South China. That sampling approach may limit the generalizability of the findings to other populations, such as older adults, non-student youth, or individuals with lower educational levels. Moreover, university students in China may have distinctive patterns of social media use and mental health experiences that may not reflect those of the broader population. Voluntary participation may also introduce self-selection bias, because individuals who chose to enroll might differ systematically from non-participants in terms of interest in mental health or social media use, potentially influencing the observed effect sizes. Future research is encouraged to replicate the findings using more representative and diverse samples, for example, community-based samples or cross-cultural comparisons.

Second, the reliance on self-report measures raises the possibility of common method bias and social desirability bias. Although the Harman single-factor test suggested that common method bias was not a significant concern in the present study, and the three-wave longitudinal design with six-month intervals reduced the impact of same-source bias, self-report measures remain an inherent limitation. Moreover, some items on the AQ short form assessing social functioning may conceptually overlap with aspects of depression or self-esteem. Thus, we controlled for baseline depression in all mediation analyses, which helped to partial out variance shared between autistic trait measures and depressive symptoms. In addition, self-reported usage duration may be subject to recall bias and may not perfectly correspond to objective usage data. However, the primary predictors in our study were not usage duration per se, but rather the frequency and pattern of active versus passive use behaviors, which are less susceptible to recall inaccuracies than absolute time estimates. Future research could further strengthen causal inference by incorporating multiple informants, for example, peer or caregiver reports, or by complementing self-reports with objective usage data, such as platform-provided screen time metrics.

Third, the measurement of TikTok use was based on an adapted scale. While the scale demonstrated good internal consistency and satisfactory construct validity via confirmatory factor analyses, a full psychometric validation study, including evaluation of its factor structure, test–retest reliability, and convergent/discriminant validity, was beyond the scope of the current investigation. More extensive validation, such as tests of measurement invariance across different populations or time points, was also not conducted. Future research would benefit from a more rigorous psychometric evaluation of the adapted scale, particularly across different cultural contexts.

Fourth, the staggered measurement design, while effective in reducing participant burden and attrition, imposed certain analytical constraints. Because autistic traits and self-esteem were measured only at T2, we were unable to control for their T1 levels. This design precluded full autoregressive control and means that we cannot fully disentangle whether the observed mediation reflects change in these constructs induced by TikTok use versus stable individual differences, nor can we completely rule out reverse causation. Furthermore, because both mediators were measured at the same time point, the assumed temporal sequence between autistic traits and self-esteem could not actually be tested empirically. The proposed serial order is therefore theoretically derived, grounded in sociometer theory (Leary, 2005) and the established link between socio-cognitive difficulties and self-evaluation, rather than empirically established by our data. Similarly, the staggered design constrained our use of structural equation modeling to comprehensively test bidirectional or reciprocal relationships between TikTok use, autistic traits, self-esteem, and depression. Finally, although the observed effect sizes were small to moderate, they are within the range typically reported in longitudinal research on media use and mental health. Nevertheless, we acknowledge that the modest magnitude of the effects means that the findings should be interpreted with caution. The practical significance of these findings remains preliminary, and future research should seek to replicate and extend these results before drawing definitive conclusions. Future research adopting fully crossed longitudinal designs, which involve measuring all constructs at all waves, could employ SEM to more thoroughly examine these dynamic relationships and enable autoregressive controls.

Fifth, the COVID-19 pandemic context, which we emphasized as a distinctive research setting, may also have acted as a confounder. The unique social restrictions during the pandemic may have influenced both TikTok use patterns and mental health outcomes, and our findings may not fully generalize to non-pandemic periods. In addition, the repeated administration of TikTok-related questionnaires across three waves may have led participants to infer the study hypotheses and adjust their responses accordingly, potentially influencing the observed associations. Although the six-month intervals between assessments likely reduced the salience of the study purpose, the possibility of demand characteristics cannot be entirely ruled out. Future research conducted under more normative social conditions would help establish the generalizability of the observed relationships.

Sixth, while we drew on several theoretical frameworks, including empathizing–systemizing theory and sociometer theory, to derive our hypotheses, we acknowledge that some of the mechanisms we discussed, for example, attentional bias, mentalizing deficits, and systemizing tendencies, were not directly measured in the present study. These mechanisms were proposed as theoretical explanations for the observed pathways, drawn from the existing literature, rather than as empirically tested constructs. In a broader context, the observed relationships between TikTok use and depression may not operate in isolation from the wider digital information ecosystem. Contemporary short-video platforms are characterized by algorithmic content curation and the proliferation of emotionally charged content that may exert differential effects on vulnerable users. Prior research has suggested that exposure to misleading or anxiety-provoking content can shape users’ affective responses and cognitive processing (Ivančík & Andrassy, 2024), and that algorithmic architectures may render users susceptible to what has been conceptualized as “fake newsworthy events” (Zakharchenko et al., 2021). These broader considerations extend beyond the scope of the current investigation, but they highlight potentially important directions for future research. Specifically, future research could further examine how individual vulnerabilities interact with systemic features of the information ecosystem, for example, algorithmic amplification of emotionally charged content, to exacerbate or mitigate the observed effects. Such an integrative approach would bridge micro-level behavioral psychology with macro-level digital sociology, offering a more comprehensive understanding of the mental health implications of social media use in the contemporary digital landscape. Future research should also employ direct measures of these processes to test the proposed mechanisms more rigorously.

## 6. Implications

Acknowledging the aforementioned limitations, the study offers several contributions to the understanding of short-video use and mental health in the post-pandemic era.

First, by examining active and passive short-video use within a longitudinal framework, the study delineates differential pathways through which these behaviors relate to depression via self-esteem and autistic traits. These findings provide preliminary evidence that the indirect effects of short-video use—whether mediated by self-esteem, autistic traits, or their serial combination—remain relevant beyond the pandemic period. As short-video platforms have become deeply embedded in daily digital life, their algorithmic logic, content ecosystems, and interactive patterns may continue to shape users’ self-perception and social cognition. Our findings may inform future efforts to understand how these platforms influence mental health, particularly by distinguishing between direct and indirect pathways.

Second, the findings retain practical relevance for the current social context in China. Intermittent calls to reduce gatherings and the persistent social constraints experienced by vulnerable groups, such as left-behind children and empty-nesters, suggest that the observed patterns may extend beyond the pandemic period. For the general public and educators, the results highlight the importance of awareness regarding the mental health risks associated with passive, immersive browsing during periods of constrained social activity. Encouraging more creative and connection-oriented active use may be a useful approach. For interventions targeting vulnerable groups, the identification of autistic traits and self-esteem as specific mediating mechanisms provides preliminary targets for future program development. Digital literacy or psychological intervention programs could consider addressing these mechanisms, though further research is needed to establish their practical utility.

Third, the validated model suggests that effective strategies may need to differentiate between the direct effects of usage behaviors and their indirect effects through mediating variables. Maintaining a moderate level of active usage while limiting passive consumption, and targeting improvements in factors such as autistic traits and self-esteem, may be promising directions for future intervention research. These insights may inform efforts to promote mental health among various populations in a post-pandemic context, though the practical applicability of these findings requires further empirical testing.

## 7. Conclusions

The present study examines the relationship between different TikTok use behaviors and depression among Chinese university students, extending prior research by demonstrating that the effects of short-form video use vary by usage mode. Our findings further clarify the underlying mechanisms through which TikTok use predicts depressive symptoms. Specifically, the mediating roles of autistic traits and self-esteem explain why active and passive use exert differential effects: active use was partially mediated by self-esteem but not by autistic traits, whereas passive use operated through both independent and sequential mediation involving autistic traits and self-esteem. These results reveal three key contributions to the literature.

First, the study provides longitudinal evidence distinguishing the differential mediation pathways for active versus passive use. The three-wave design, with six-month intervals separating predictors, mediators, and outcomes, offers stronger support for the proposed directional pathways than cross-sectional or two-wave designs. The findings clarify that passive use operates through three indirect pathways, specifically via autistic traits, self-esteem, and their sequential mediation, whereas active use operates only through self-esteem. This nuanced pattern would not have been captured without the longitudinal design.

Second, the study extends the active–passive social media use framework by introducing autistic traits as a novel mediating mechanism. Prior research in this tradition has focused predominantly on mediators such as social comparison, self-esteem, and fear of missing out. By demonstrating that passive TikTok use predicts depression through elevated autistic traits, whereas active use does not, the study opens a new theoretical direction that links media use behaviors to socio-cognitive vulnerabilities. This contribution is particularly significant given that autistic traits have rarely been examined as outcomes of media use in general population samples.

Third, the findings yield actionable implications for intervention. For individuals who predominantly engage in passive, immersive browsing, reducing algorithmic-driven consumption and addressing socio-cognitive vulnerabilities may be key intervention targets. For those who use TikTok actively, fostering self-esteem through positive self-expression and meaningful social interaction may be more beneficial. The identification of autistic traits and self-esteem as specific mediating mechanisms provides precise targets for digital literacy programs and psychological interventions.

The present study thus contributes to the active–passive social media literature by demonstrating that distinct usage patterns operate through fundamentally different psychological pathways. This finding underscores the importance of moving beyond the active–passive dichotomy in intervention design and tailoring strategies to the specific mechanisms activated by different forms of engagement.

## Figures and Tables

**Table 1:** Descriptive statistics and correlations between variables.

Variables	1	2	3	4	5	6
1. T1 Active TikTok use	—					
2. T1 Passive TikTok use	−0.13 **	—				
3. T1 Depression	−0.18 ***	0.24 ***	—			
4. T2 Autistic traits	−0.13 **	0.43 ***	0.22 ***	—		
5. T2 Self-esteem	0.42 ***	−0.51 ***	−0.27 ***	−0.56 ***	—	
6. T3 Depression	−0.30 ***	0.36 ***	0.52 ***	0.41 ***	−0.53 ***	—
Mean	9.93	11.79	9.46	44.74	24.68	8.38
SD	4.65	4.68	5.08	15.13	5.66	4.76

Note. *N* = 590. ** *p* < 0.01. *** *p* < 0.001.

**Table 2:** Mediating effects of autistic traits and self-esteem on the relationship between TikTok use and depression.

Regression Equation		Significance of Regression Coefficients				Bootstrap	
Outcome Variables	Independent Variables	β	*SE*	*t*	*p*	LLCI	ULCI
T3 Depression	Gender	0.01	0.03	0.28	0.78	−0.06	0.08
	Age	−0.01	0.04	−0.20	0.84	−0.08	0.06
	Duration of TikTok use	−0.02	0.03	−0.48	0.63	−0.08	0.05
	T1 Depression	0.43 ***	0.05	8.83	<0.001	0.33	0.52
	T1 Active TikTok use	−0.20 ***	0.04	−5.25	<0.001	−0.27	−0.12
	T1 Passive TikTok use	0.23 ***	0.04	5.77	<0.001	0.15	0.31
T2 Autistic traits	Gender	−0.002	0.04	−0.04	0.97	−0.08	0.07
	Age	−0.01	0.04	−0.40	0.69	−0.09	0.06
	Duration of TikTok use	0.08 *	0.04	2.21	<0.05	0.01	0.16
	T1 Depression	0.11 *	0.05	2.39	<0.05	0.02	0.21
	T1 Active TikTok use	−0.05	0.04	−1.38	0.17	−0.13	0.02
	T1 Passive TikTok use	0.38 ***	0.05	8.14	<0.001	0.29	0.48
T2 Self-esteem	Gender	0.03	0.03	1.17	0.24	−0.02	0.09
	Age	0.02	0.03	0.62	0.54	−0.04	0.07
	Duration of TikTok use	−0.01	0.03	−0.19	0.85	−0.06	0.05
	T1 Depression	−0.06	0.04	−1.61	0.11	−0.13	0.01
	T1 Active TikTok use	0.32 ***	0.03	9.32	<0.001	0.25	0.39
	T1 Passive TikTok use	−0.29 ***	0.04	−7.56	<0.001	−0.37	−0.22
	T2 Autistic traits	−0.38 ***	0.04	−10.14	<0.001	−0.45	−0.30
T3 Depression	Gender	0.02	0.03	0.64	0.52	−0.04	0.08
	Age	0.002	0.03	0.06	0.95	−0.06	0.06
	Duration of TikTok use	−0.04	0.03	−1.17	0.24	−0.10	0.03
	T1 Depression	0.38 ***	0.05	8.47	<0.001	0.30	0.47
	T1 Active TikTok use	−0.09 *	0.03	−2.55	<0.05	−0.16	−0.02
	T1 Passive TikTok use	0.06	0.04	1.56	0.12	−0.01	0.13
	T2 Autistic traits	0.13 **	0.05	2.67	<0.01	0.03	0.22
	T2 Self-esteem	−0.29 ***	0.05	−5.39	<0.001	−0.40	−0.19

Note. *N* = 590. Bootstrap sample size = 5000. LL = low limit, CI = confidence interval, UL = upper limit. * *p* < 0.05. ** *p* < 0.01. *** *p* < 0.001.

**Table 3:** Total, direct, and indirect effects.

Behaviors	Effects	Coefficients	*SE*	Bootstrap	
				LLCI	ULCI
Active TikTok use	Total effect	−0.20 ***	0.04	−0.27	−0.12
	Direct effect	−0.09 *	0.03	−0.16	−0.02
	Mediating effect of autistic traits	−0.01	0.01	−0.02	0.001
	Chain mediating effect	−0.01	0.004	−0.02	0.002
	Mediating effect of self-esteem	−0.09 ***	0.02	−0.14	−0.05
Passive TikTok use	Total effect	0.23 ***	0.04	0.15	0.31
	Direct effect	0.06	0.04	−0.01	0.13
	Mediating effect of autistic traits	0.05 **	0.02	0.01	0.09
	Chain mediating effect	0.04 ***	0.01	0.02	0.07
	Mediating effect of self-esteem	0.09 ***	0.02	0.05	0.13

Note. *N* = 590. Bootstrap sample size = 5000. *SE* = standard error. LL = low limit, CI = confidence interval, UL = upper limit. * *p* < 0.05. ** *p* < 0.01. *** *p* < 0.001.

## Data Availability

The data are not publicly available due to privacy and ethical restrictions. The data that support the findings of this study are available on reasonable request from the corresponding author following the completion of a privacy and fair use agreement.
